# Development and implementation of a novel clinical teaching model integrating real-world case with patient-specific three-dimensional imaging in urology

**DOI:** 10.1186/s12909-026-09765-9

**Published:** 2026-07-01

**Authors:** Cong Zhang, Mohammad Rasool Ghiasi, Jinhuan Cheng, Haocheng Cui, Guanghao Li, Qinquan Sha, Dongshan Chen, Xiang Zhang

**Affiliations:** 1 Department of Urology, Qilu Hospital of Shandong University, Jinan, 250014 China; 2 Department of Electronic Engineering, Tsinghua University, Beijing, 100084 China; 3 Department of education, Qilu Hospital of Shandong University, Jinan, 250014 China

**Keywords:** Urological clinical practicum, Real-world case-based learning, Three-Dimensional imaging

## Abstract

**Background:**

Urology encompasses a diverse array of pathologies with complex clinical presentations, demanding rigorous hands-on clinical practice and high-quality pedagogical training for medical interns. To overcome the limitations of traditional teaching models, which typically combines theoretical instruction with ward observation often provide limited exposure, we developed and validated a novel clinical teaching model that is based on real-world clinical cases in individualized three-dimensional imaging and evaluated its feasibility and effectiveness in urological internship teaching through prospective experiments.

**Objective:**

To evaluate the feasibility and effectiveness of a novel clinical teaching model that integrates real-world clinical cases with individualized three-dimensional imaging in urology interns.

**Methods:**

During the 2024–2025 academic year, 128 interns in the Department of Urology were randomly assigned to two groups based on student ID numbers: the traditional teaching mode (TTM) arm and the new teaching mode (NTM) arm, with 64 interns in each group. The TTM group received the conventional approach of thematic theoretical teaching alongside clinical case observation, while the NTM arm implemented the novel approach. After a 2-week internship, the educational effects of trainees were assessed by confidential questionnaire, theoretical knowledge examination, and clinical practice assessments, followed by statistical analysis of the results.

**Results:**

Interns in the NTM group outperformed those in the TTM group (*p* < 0.05). Although some specific item assessments revealed no significant differences between the two groups, the overall scores of interns in the NTM group on confidential questionnaires, theoretical knowledge examinations, and clinical practice assessments were significantly higher than those in the TTM group (*p* < 0.05). The weighted composite score (confidential questionnaire constituted 0.2, theoretical knowledge examination constituted 0.4, clinical practice examination constituted 0.4) of the NTM group was significantly higher (79.75 ± 3.91 vs. 85.30 ± 6.03, *p* < 0.001).

**Conclusion:**

This study indicates that the NTM is well accepted by interns and those receiving clinical teaching through the NTM show superior theoretical knowledge and clinical skills over the TTM.

**Supplementary Information:**

The online version contains supplementary material available at 10.1186/s12909-026-09765-9.

## Introduction

Urology, a fundamental surgical specialty, spans a wide range of diseases, complex pathogenesis, and significant individual differences in patients’ clinical manifestations [1]. Consequently, urology interns must master theoretical knowledge and undergo rigorous clinical training. The primary goal of undergraduate urology internship is to merge these two elements, integrating theory with practice to cultivate future urologists who can meet the evolving health care demands. However, traditional teaching methods (TTM) often follow a segmented approach, primarily consisting of “classroom PPT theoretical instruction followed by clinical case discussions”. This teacher-centered model restricts learning to classrooms settings and emphasizes rote memorization and examination preparation, leading to a disconnect between theory and practice. Consequently, student motivation declines, resulting in ineffective teaching outcomes [2, 3]. Therefore, it is essential to explore a novel teaching model that enhances learning efficiency and bridges urological theory and practice.

The innovative teaching method uses real-world cases, conducted with full patient and family consent, which immerse students in the frontline of clinical practice. This approach draws on the patient’s current condition to intuitively explain how clinical manifestations reflect the underlying theoretical knowledge and pathogenesis through real-time communication and examination results while students simultaneously develop critical thinking skills for differential diagnosis. This model enables students to gain a deeper grasp of the disease characteristics and progression, adapt more rapidly to the clinical working environment and establish a solid foundation for their future clinical roles.

Three-dimensional reconstruction imaging is produced by advanced processing of two-dimensional CT scans [4]. Although two-dimensional CT was capable of showing lesions and provide important information (such as benign and malignant tumors, hardness of stones, etc.), but the intra-organs location of lesions (such as tumors, stones, etc.) is poorly visualized (for example, the distribution of renal tumors in the kidney is not completely visualized) since it only provides a single-plane view. In addition, anatomy of blood vessels in organs and tissues can differ among individuals, such as the number and specific route of renal arteriovenous vessels. These limitations are overcome by the use three-dimensional imaging which accurately localizes the lesion and depicts vascular anatomy, thus enabling precise lesion resections and preventing accidental vessel damage, reducing hemorrhage [5], Proving to be crucial for surgical planning and execution [6]. Moreover, three-dimensional images clearly depict the anatomical structures of lesion tissues, aiding interns, who may have limited anatomical knowledge, in understanding the lesion -tissue relationships, as well as enhancing their grasp of course of disease and surgical treatment principles.

Over the past decade, undergraduate surgical training has witnessed a major pedagogical paradigm shift driven by Case-Based Learning (CBL) [7] and Simulation-Based Medical Education (SBME) [8]. Traditional lecture-based rounds often struggle to cultivate spatial reasoning, which is critical for comprehending complex surgical anatomy. Recent educational validation studies in general surgery, radiology, and urology have demonstrated that three-dimensional (3D) visualization and virtual models can significantly enhance students’ spatial cognition and diagnostic accuracy. However, most existing 3D interventions are confined to pre-clinical lecture halls or passive pre-operative viewing. The precise novelty of the present work lies in the real-time, interactive integration of patient-specific 3D digital reconstructions directly at the patient’s bedside within an active undergraduate urology internship, thereby establishing a direct cognitive link between multi-dimensional digital anatomy and live clinical presentations.

To address these educational gaps, we developed and evaluated a novel teaching model for clinical internship that combines real-world cases with patient-specific three-dimensional imaging. Clinical teaching experiments were further conducted to evaluate the feasibility and effectiveness of this model.

## Materials and methods

### Basic characteristics

A convenience sample of 128 fifth-year undergraduate interns majoring in clinical medicine from Qilu Medical College of Shandong University participated in the urology internship at Qilu Hospital of Shandong University during the 2024–2025 academic year. The group consisted of 61 males and 67 females (mean age of 21.93 ± 1.14 years). All interns had systematically studied urological theoretical knowledge prior to internship. Interns were randomly assigned to either the TTM or NTM group in a 1:1 ratio (*n* = 64 per arm). To prevent selection bias, true simple randomization was performed. An independent teaching secretary, not involved in the teaching or evaluation processes, generated a random allocation sequence using an online random number generator (www.random.org) applied to the students’ unique ID numbers. Strict allocation concealment was maintained; the master allocation list was secured in a password-protected file managed exclusively by the secretary. Group assignments remained concealed from both the students and the clinical instructors until the morning of their first day of clinical rotation. NTM utilized a new teaching method that integrated real-world cases with three-dimensional images, while the TTM followed the traditional teaching mode, which involved thematic theoretical instruction combined with clinical case observation. To eliminate instructor variability as a confounding factor, a designated core group of senior urological physicians (all with ≥ 5 years of clinical teaching experience) was responsible for cross-delivering both the TTM and NTM curricula. Prior to the study, all participating instructors underwent standardized pedagogical training on both the traditional theoretical approach and the 3D-assisted bedside teaching protocols to ensure uniform delivery. Furthermore, all external teaching factors, such as textbooks, learning environments, and syllabus objectives, were strictly standardized to ensure robust consistency between the two arms.

The reporting of this study adheres to standard guidelines for educational intervention research, drawing upon the principles of the GREET (Guideline for Reporting Evidence-based practice Educational interventions and Teaching) statement to ensure methodological transparency and reproducibility.

### development of the novel teaching model integrating real-world cases with three-dimensional imaging

**Fig. 1:**
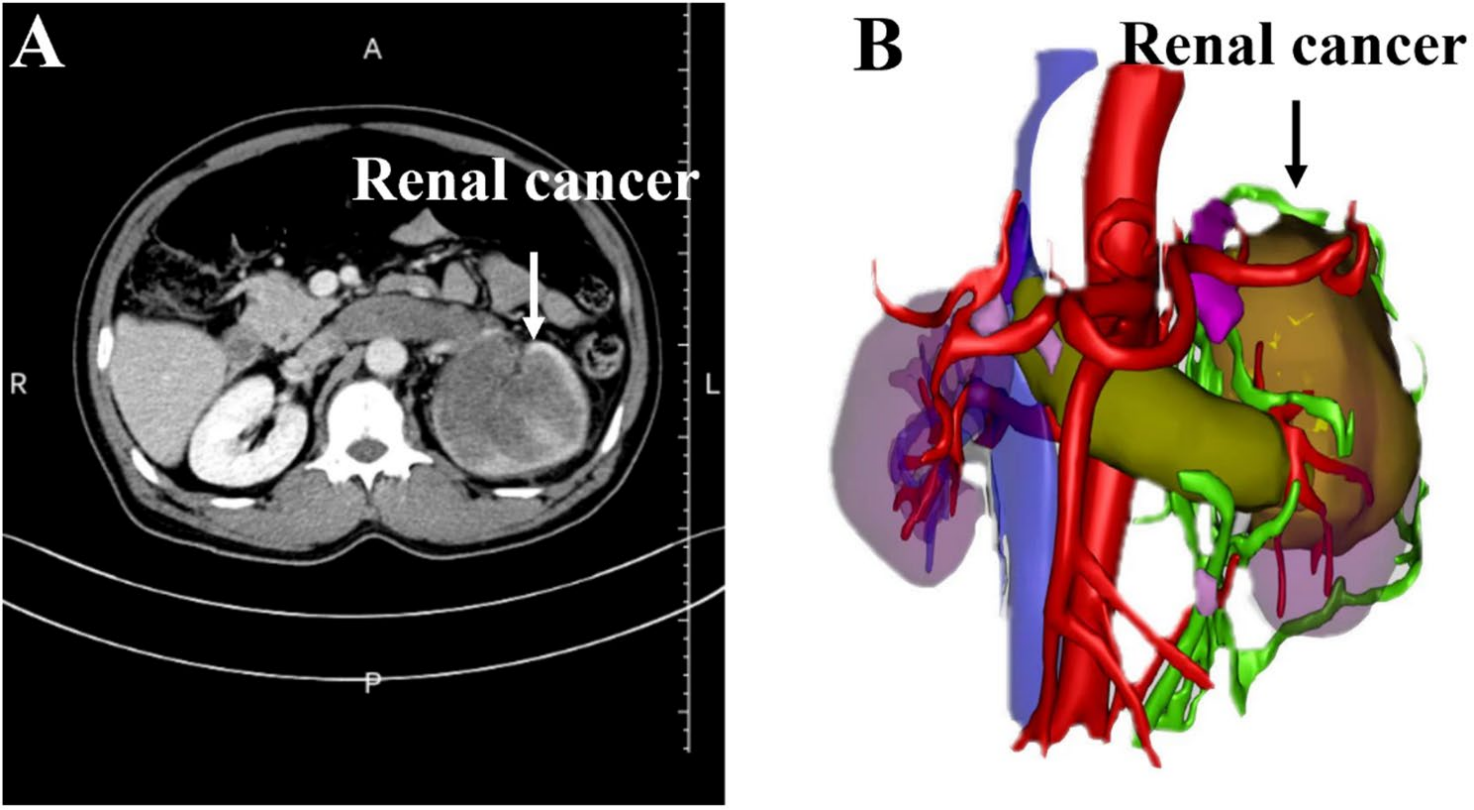
Three-dimensional image construction. **A** Patient’s specific two-dimensional CT image. **B** Patient’s specific three-dimensional image

**Fig. 2:**
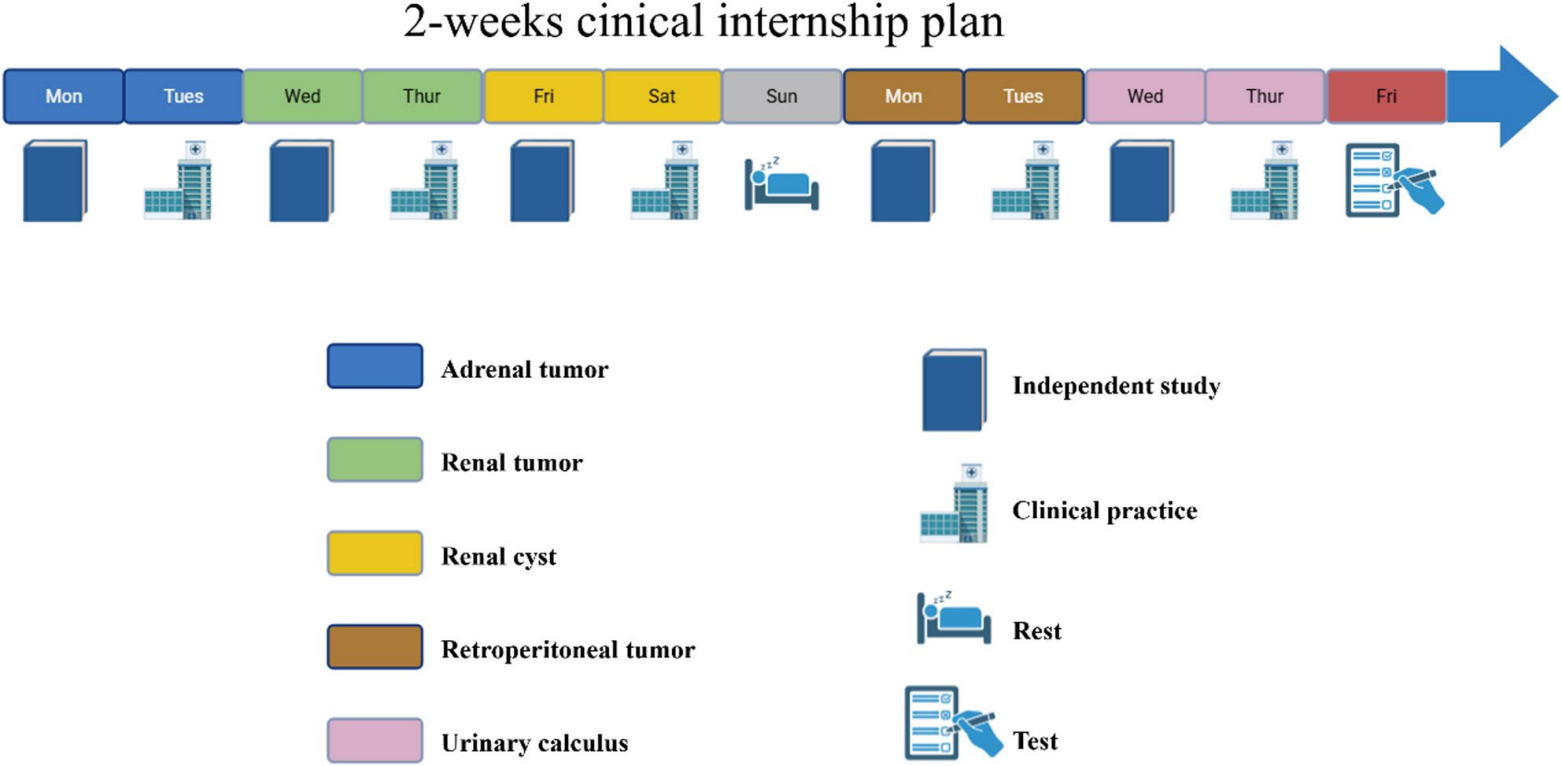
Two-week internship course arrangement

The NTM arm focused on real-world cases, integrating individualized three-dimensional imaging based on patients’ CT and other imaging data (in the case of renal tumor, Fig. 1). Five common urological diseases —adrenal tumor, renal tumor, renal cyst, retroperitoneal tumor, and urinary calculi —were selected because their characteristics and mastery their diagnosis and treatment were essential for urology interns. Each disease had a 2-day training duration, with all students required to complete a literature review 1 day prior to clinical internship. On the morning of admission, the TTM group received theoretical instruction in a demonstration classroom and engaged in clinical practice with teaching tutors in the afternoon. The NTM group began with bedside case teaching accompanied by patients’ individual three-dimensional images in the morning, led by teaching instructors, followed by clinical practice in the afternoon. On the final day of internship training, all participants first completed a subjective confidential questionnaire, then followed by a clinical practice assessment, and participated in a unified theoretical examination finally. The overall internship process is illustrated in Figure S1, with internship plan in Fig. 2.

### Evaluation of the novel teaching model

The acceptance and effectiveness of NTM were the main objectives of the present study; therefore, the assessment system was carefully designed. It consisted of two parts: (1) an confidential survey to evaluate the interns’ acceptance of each teaching model after the internship; and (2) an objective assessment of teaching effect, utilizing both theoretical and clinical practice assessments. To ensure absolute fairness and neutrality, both the theoretical and clinical examinations were strictly aligned with the standard undergraduate urology syllabus. The theoretical examination comprised multiple-choice and short-answer questions focusing on etiology, clinical manifestations, diagnostic criteria, and standard therapeutic principles, deliberately excluding highly specific spatial anatomical questions that might inadvertently favor the NTM group. The clinical practice examination assessed universal clinical competencies, including comprehensive history taking, standardized physical examinations, and basic diagnostic workflows, without requiring the interpretation of 3D models during the test.

A confidential questionnaire (Table S1) was designed to assess the interns’ learning experience and acceptance of the two teaching models. The design of the questionnaire was guided by validated principles from previous medical education literature [9, 10]. To establish content validity, the instrument underwent a rigorous review by a panel of three senior medical educators in the department prior to distribution. Furthermore, pilot testing was conducted on a separate cohort of 20 clinical interns to confirm face validity. The internal consistency of the questionnaire was subsequently evaluated, yielding an overall Cronbach’s alpha coefficient of 0.86, which indicates high reliability. To ensure honesty while allowing for comprehensive scoring, questionnaires required a 12-digit student ID. An independent teaching secretary, uninvolved in the teaching or evaluation, collected the data, linked the questionnaire scores with theoretical and clinical exam results using the student IDs, and subsequently removed all identifiers. This fully de-identified dataset was then provided to the researchers for statistical analysis, ensuring complete confidentiality. This questionnaire contains five domains: overall impression, knowledge acquisition, improvement of clinical skills, cultivation of clinical thinking, and doctor-patient communication ability. Each domain provides four response levels of satisfaction (very satisfied, good, average, and dissatisfied); levels are 5 points apart giving a maximum score of 20 points to domains, leading to a total score of 100. For the thematic practice examination, scoring was strictly based on Bloom’s Taxonomy [11], an internationally validated framework (Table S2), ensuring the standardized assessment of clinical competencies. The theoretical examination was assembled using the historically validated and standardized question bank of Qilu Medical College, to minimize assessment bias, which was graded blindly. Two independent clinicians, who were strictly blinded to the students’ identities and group allocations, reviewed and scored the de-identified examination papers with a maximum score of 100 points.

### Data analysis

To evaluate the comprehensive performance of the interns, a weighted composite score was calculated. Aligning with the primary objectives of the clinical internship, objective competencies were prioritized: theoretical knowledge and clinical practice were each assigned a weight of 40%. The subjective assessment of the teaching model via the confidential questionnaire was assigned a 20% weight. Thus, the total score for each intern was calculated as: confidential questionnaire × 0.2 + theoretical knowledge examination × 0.4 + clinical practice examination × 0.4. Data analysis was performed using SPSS (IBM Corp., Armonk, NY, USA). To accommodate the ordinal nature of the questionnaire evaluations and clinical practice rubrics, non-parametric statistical methods were systematically employed. Continuous and ordinal variables are expressed as mean ± standard deviation (SD) alongside medians and interquartile ranges (IQR) to ensure full data transparency. Group comparisons between the TTM and NTM cohorts were performed using the Mann-Whitney U test. To evaluate the stability and validity of the 20%/40%/40% composite score weighting framework, a formal sensitivity analysis was conducted by recalculating the composite scores under alternative configurations (specifically, 0%/50%/50% and 10%/45%/45% weighting models), with group differences consistently evaluated via the Mann-Whitney U test. All *p*-values are two-tailed, with statistical significance defined as *p* < 0.05. *p*-value formatting and notation have been standardized to three decimal places throughout the manuscript (with values less than 0.001 expressed uniformly as *p* < 0.001). To demonstrate the magnitude and educational relevance of the findings, the 95% confidence intervals (CIs) for the mean differences between the two groups were calculated. Additionally, effect sizes were reported using Cohen’s d, with thresholds of 0.2, 0.5, and 0.8 defined as small, medium, and large effects, respectively.

## Results

### Participants

**Fig. 3:**
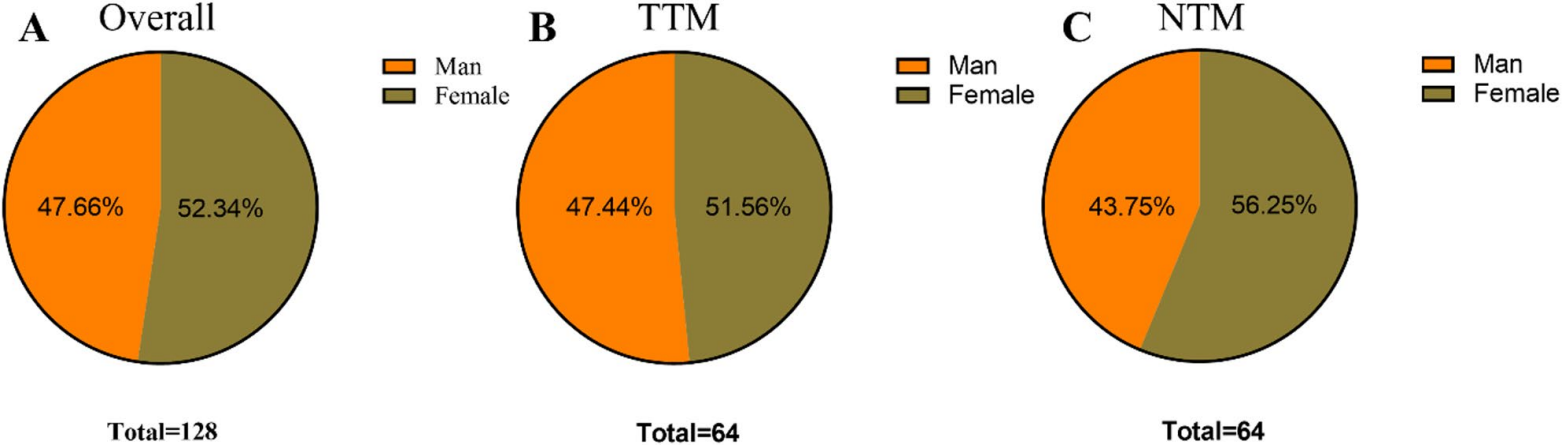
Gender distribution of medical interns. **A** Overall cohort. **B** TTM group. **C** NTM

All 128 fifth-year clinical medicine undergraduates from Qilu Medical College of Shandong University participated in clinical practice at the Department of Urology during the 2024–2025 academic year (61 males, 67 females; mean age 21.93 ± 1.14 years). These interns were randomly divided into TTM (*n* = 64) and NTM (*n* = 64). The TTM group comprised 33 males and 31 females, with a mean age of 21.89 ± 1.01 years, whereas the NTM group consisted of 28 males and 36 females, with a mean age of 21.80 ± 1.13 years. No significant statistical differences were found in age or sex distribution of arms (Table 1; Fig. 3).

**Table 1:** The basic characteristics of all the participants

Item	TTM(*n* = 64)	NTM(*n* = 64)	*p*
Gender			0.479
Man	33	28	
Female	31	36	
Age	21.89 ± 1.01	21.80 ± 1.13	0.62
Overall score	79.75 ± 3.91	85.30 ± 6.03	< 0.001

The “Mann-Whitney U” was used for statistical analysis, and *p* < 0.05 was considered statistically significant

### Acceptance

**Fig. 4:**
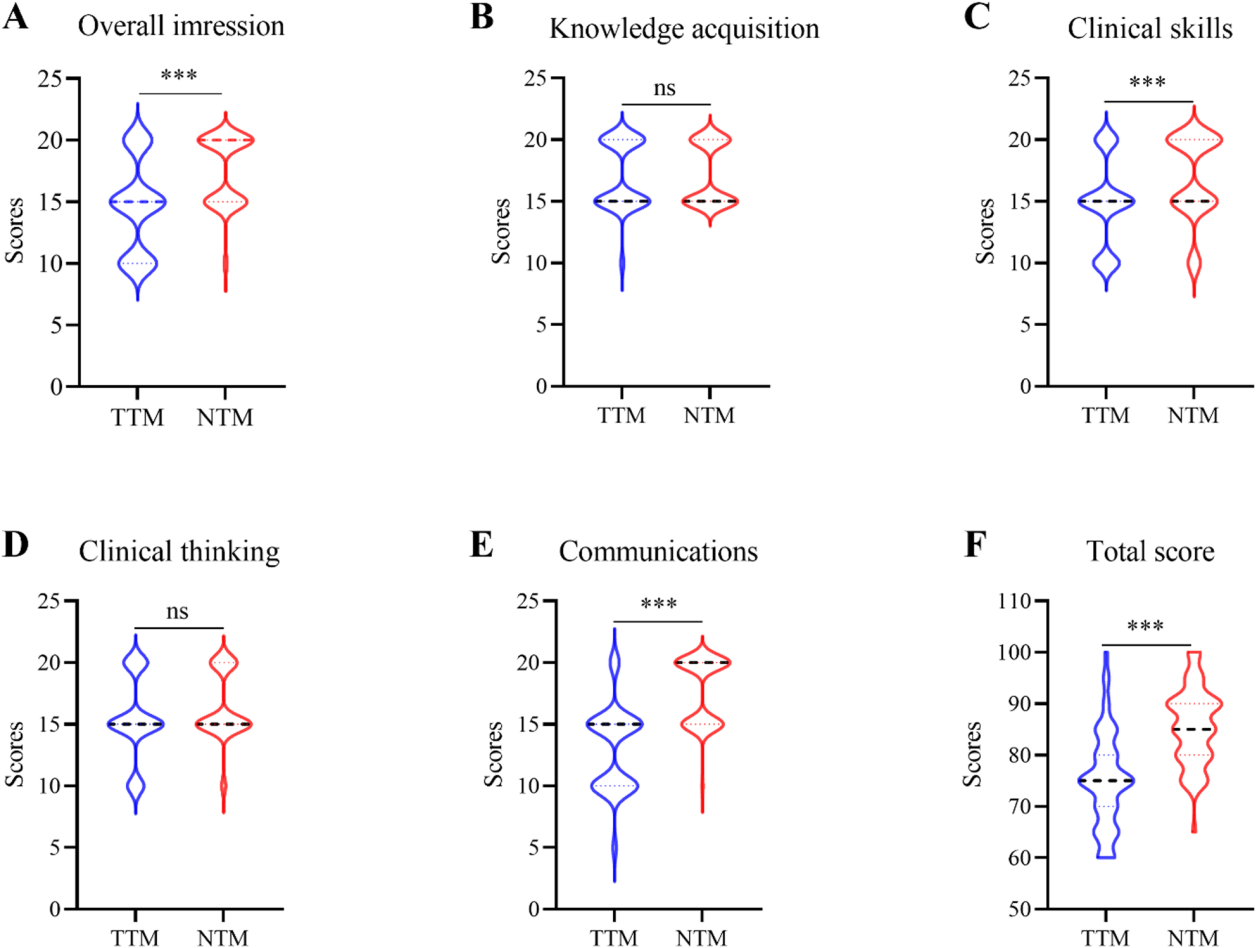
Distribution of confidential questionnaire domains. Data are presented as box-and-whisker plots capturing the median, interquartile range (IQR), and full distribution range of intern evaluations across (**A**) overall impression, (**B**) knowledge acquisition, (**C**) clinical skills, (**D**) clinical thinking, and (**E**) communication. **F** Overall composite score. ns: no statistical significance, ***p < 0.001

We designed an confidential questionnaire focusing on five aspects: overall impression, knowledge acquisition, improvement of clinical skills, cultivation of clinical thinking, and doctor-patient communication ability to evaluate the interns’ acceptance of TTM and NTM. Each aspect was rated out of 20 points, with five grades differing by 5 points (0, 5, 10, 15, and 20), leading to a total score of 100 points. For overall impression, interns rated NTM significantly higher than TTM (*p* < 0.001). Regarding knowledge acquisition and clinical thinking, both TTM and NTM scored similarly (*p* > 0.050). However, for the improvement of clinical skills and doctor-patient communication ability, NTM surpassed TTM (*p* < 0.05). The results indicated a higher acceptance of interns for the NTM than TTM (TTM: 74.92 ± 9.19 vs. NTM: 85.63 ± 7.48, *p* < 0.001; Table 2; Fig. 4).

**Table 2:** The results of the anonymous subjective questionnaire

Item	TTM(*n* = 64) Median/Mean ± SD	NTM(*n* = 64) Median/Mean ± SD	*p*
General impression	15/14.61 ± 3.71	20/17.58 ± 2.82	< 0.001
Knowledge acquisition	15/17.03 ± 2.78	15/17.11 ± 2.49	> 0.050
Clinical skills	15/14.84 ± 3.45	15/16.88 ± 3.39	0.001
Clinical thinking	15/15.39 ± 3.25	15/16.33 ± 2.71	0.105
Communication	15/13.05 ± 3.41	20/17.73 ± 2.66	< 0.001
Overall score	75/74.92 ± 9.19	85/85.63 ± 7.48	< 0.001

The “Mann-Whitney U” was used for statistical analysis, and *p* < 0.05 was considered statistically significant

### Clinical-practice examination

**Fig. 5:**
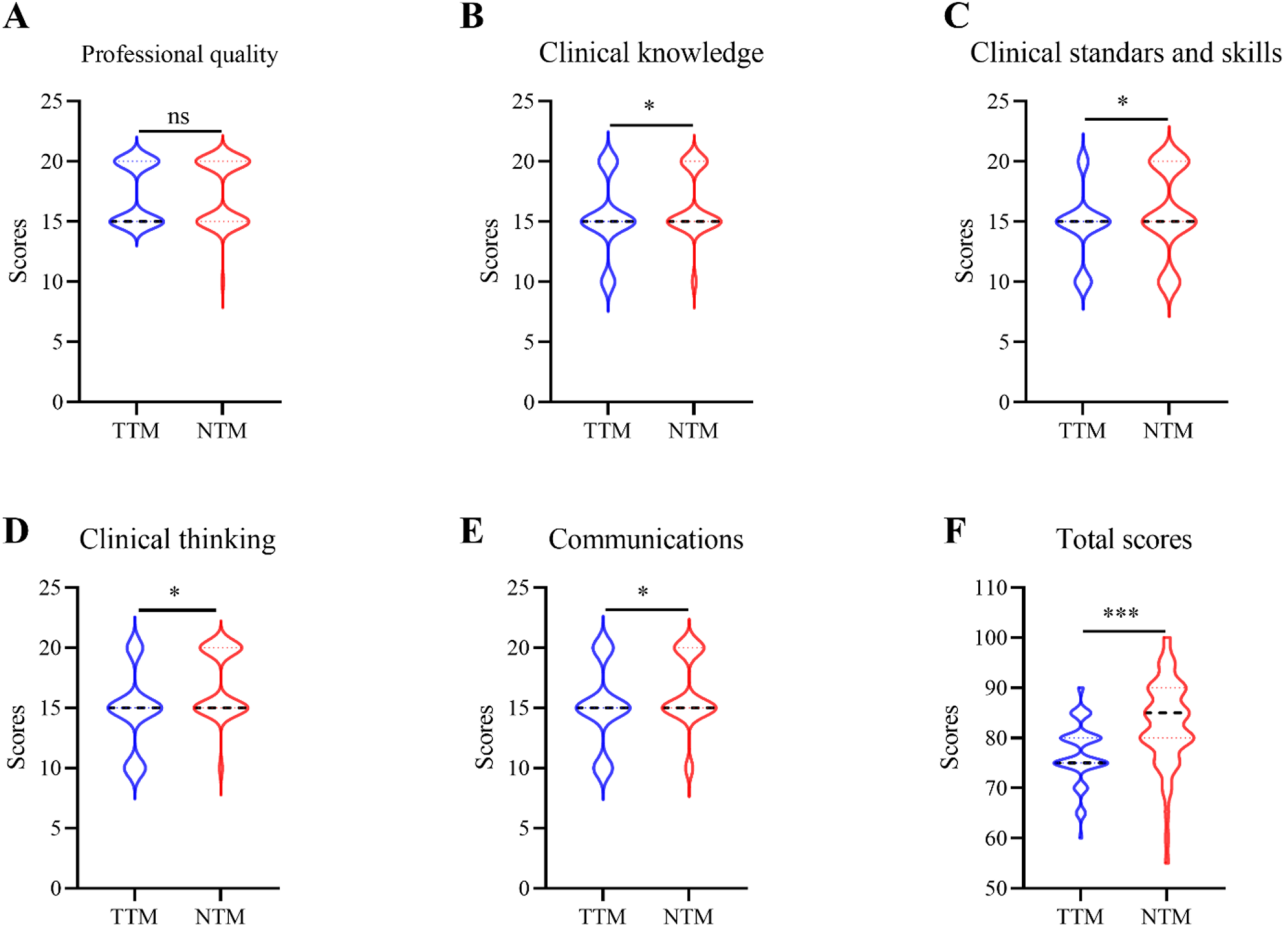
Distribution of clinical practice assessment scores. Box-and-whisker plots displaying the data spread and median lines for (**A**) professional quality, (**B**) clinical knowledge, (**C**) clinical standards and skills, (**D**) clinical thinking, and (**E**) communications. **F** Total clinical practice score. *p < 0.05, ***p < 0.001

We evaluated clinical competency of participants of both models across five domains: professional quality, knowledge acquisition and clinical skill improvement, cultivation of independent clinical thinking, promotion of interpersonal communication, and self-improvement. Professional quality did not differ between TTM and NTM (*p* = 0.724), with both achieving satisfactory scores, suggesting improvement of professionalism across both arms. However, in terms of clinical standards and skills, cultivation of independent clinical thinking, promotion of interpersonal communication, and self-improvement, the NTM demonstrated superior teaching effects compared to the TTM. Although the NTM cohort achieved a higher descriptive score in clinical knowledge, this sub-domain did not achieve statistical significance under non-parametric testing (*p* = 0.066). Notably, in clinical thinking, interns taught using NTM performed significantly better than those taught with TTM (TTM: 14.69 ± 3.20 vs. NTM: 16.95 ± 2.76, *p* < 0.001). Overall, the clinical practice scores favored NTM (TTM: 76.95 ± 6.08 vs. NTM: 82.73 ± 8.68, *p* < 0.001) (Table 3; Fig. 5).

**Table 3:** The scores of the departmental rotation examination of all the participants

Item	TTM(*n* = 64) Median/Mean ± SD	NTM(*n* = 64) Median/Mean ± SD	*p*
Professionalism	15/17.27 ± 2.51	17.5/17.42 ± 2.67	0.724
Clinical knowledge	15/15.31 ± 3.07	15/16.33 ± 2.71	0.066
Clinical standards and skills	15/14.61 ± 2.86	15/15.78 ± 3.59	0.031
Clinical thinking	15/14.69 ± 3.20	15/16.95 ± 2.76	< 0.001
Communication	15/15.08 ± 3.27	15/16.25 ± 2.96	0.046
Overall score	75/76.95 ± 6.08	85/82.73 ± 8.68	< 0.001

The “Mann-Whitney U” was used for statistical analysis, and *p* < 0.05 was considered statistically significant

### Theoretical examination

**Fig. 6:**
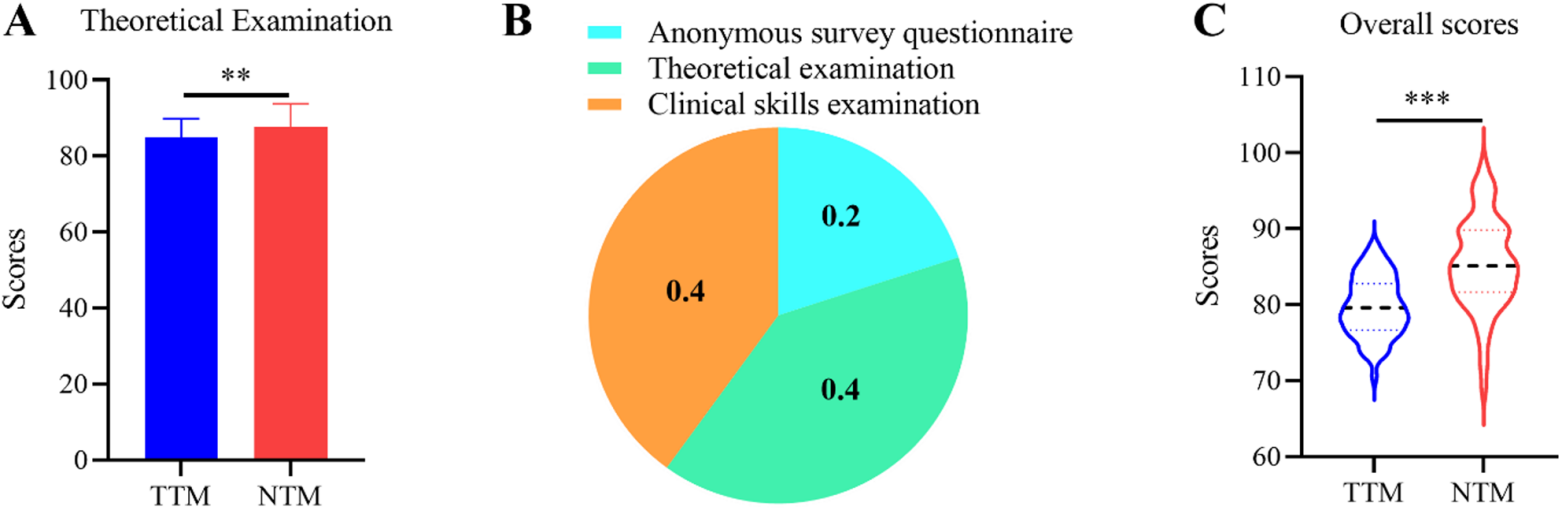
Theoretical and composite scores. **A** Theoretical examination scores. **B** Weighting components of the overall composite score. **C** Overall composite scores. **p < 0.01, ***p < 0.001

We evaluated theoretical knowledge with a unified examination. NTM students achieved 87.70 ± 5.99, significantly higher than TTM students (84.95 ± 4.83, *p* = 0.005) (Table 4; Fig. 6).

**Table 4:** Theoretical examination

Item	TTM(*n* = 64)	NTM(*n* = 64)	*p*
Basic knowledge	84.95 ± 4.83	87.70 ± 5.99	0.005

The “Mann-Whitney U” was used for statistical analysis, and *p* < 0.05 was considered statistically significant

### Overall composite performance evaluation

The non-parametric analysis demonstrated that medical interns in the NTM group achieved higher overall performance scores compared to those in the TTM group. NTM median: 85.10 (IQR:89.80-81.65) vs. TTM median: 79.60 (IQR: 82.75–76.65), *p* < 0.001). The mean difference between the two cohorts was 5.55 (95% CI: 3.77–7.33), representing a large and educationally meaningful effect size (Cohen’s d = 1.09). (Table 1; Fig. 6). Crucially, the formal sensitivity analysis confirmed the high stability of this finding; when alternative weighting configurations were tested (recalculating the scores under 0%/50%/50% and 10%/45%/45% models), the NTM cohort consistently and significantly outperformed the TTM group (*p* < 0.001 across all models), proving that the central conclusion is entirely robust and insensitive to the specific mathematical weights chosen. However, the magnitude of improvement was not uniform across all evaluated competency streams. While higher-order competencies like clinical thinking exhibited substantial, robust enhancements, the differences observed in basic communication sub-domains were comparatively modest, suggesting varying degrees of pedagogical impact across different skill sets.

## Discussion

In this study, we enrolled 128 fifth-year undergraduate interns who practiced clinically in the Department of Urology at Qilu Hospital of Shandong University during the 2024–2025 academic year. They had completed anatomy, pathology, surgery and urology courses of the first four years of their studies and had finished their clinical clerkship in their fourth year. Consequently, they possessed a solid theoretical foundation and basic understanding of urological diseases. Fifth year internship focus to deepen their understanding of clinical work, promote the integration of theoretical knowledge with clinical practice, and establish a strong foundation for their future residency.

The traditional clinical internship teaching model involves senior clinical doctors, who have extensive experience, provide thematic theoretical lessons in a classroom setting before advancing ward rounds to perform patient inquiries, physical examinations, and to formulate examination and treatment plans for corresponding diseases [12]. Although this teaching model has been in place for decades and has a relatively refined system [13], individual differences among patients and varying stages of disease progression often lead to discrepancies between theoretical teachings and the actual clinical manifestations, imaging, and inspection results observed in patients. Such variations can hinder interns to apply theoretical knowledge to practical application.

In contrast, the novel teaching model (NTM) still retains clinical teachers but allows interns to directly engage with patients in the ward who present with diseases relevant to their learning without the initial classroom phase. After communication with patients and their families, and upon obtaining consent, bedside teaching begins. For instance, in renal tumor cases, three-dimensional reconstruction technology generates individualized images based on the patient’s imaging results (CT or MRI), detailing anatomical features such as tumor size, kidney location, and blood vessel pathways [14]. This provides critical reference points to formulate preoperative strategies and select intraoperative approaches [15]. Personalized three-dimensional images, along with the patient’s clinical manifestations and examination results, clinical teaching physicians present a systematic overview of the disease’s occurrence and progression, anatomical characteristics, pathological mechanisms, and detailed surgical plans. Compared to TTM, this novel approach effectively bridges theoretical knowledge with clinical practice, offering clearer and more systematic analysis of disease processes in real-world contexts. This helps interns to improve their understanding of urological diseases and achieve their clinical learning objectives in urology more efficiently and with higher quality.

While the application of 3D imaging in medical education is extensively documented, previous literature has primarily focused on its utility in preclinical anatomy courses using generic models. More broadly, contemporary urological educational research has increasingly focused on tracking and optimizing learner engagement with such innovative digital modalities, demonstrating that interactive media formats and specialized digital platform designs are critical determinants of student interaction patterns and pedagogical efficacy [16]. The distinct novelty and pedagogical value of our NTM lie in its integration of patient-specific 3D models directly at the bedside within the context of real-world clinical cases. Rather than merely serving as a spatial visualization tool, the individualized 3D models in our study act as a catalyst for higher-order clinical competencies. By visualizing the exact pathology of the patient they are actively interviewing, interns seamlessly bridge the gap between abstract pathological concepts and tangible clinical decision-making, such as individualized surgical planning. Furthermore, this study demonstrates the unique value of patient-specific 3D models as a medium for doctor-patient communication. We observed that utilizing these images as visual aids empowered interns to intuitively explain complex surgical concepts to patients and their families, which accounts for the significantly higher communication scores in the NTM cohort. Therefore, this study adds to the current body of knowledge by validating a structured pedagogical shift: moving 3D technology from preclinical anatomical memorization to a dynamic tool that cultivates advanced clinical reasoning and humanistic communication in final-year medical interns.

Upon completing the 2-week internship in urology, all interns participated in unified theoretical and practical assessments, along with confidential questionnaires to evaluate the feasibility and teaching quality of the two models. The evaluation criteria were divided into three weighted parts: confidential questionnaires (0.2), thematic theoretical examinations (0.4), and thematic practical examinations (0.4), each with a maximum score of 100 points. Finally results indicated that interns utilizing the NTM outperformed those adopting TTM in multiple indicators.

From the perspective of the weighted composite scores, interns in the NTM arm demonstrated significantly better performance than those in the TTM arm, suggesting that the novel teaching method more effectively enhances the clinical learning quality of interns.

Analysis of the confidential questionnaire content revealed that interns rated the new teaching plan more favorably regarding their overall impression of the teaching mode, indicating it provided better learning experience. In terms of knowledge acquisition, interns expressed satisfaction with both NTM and TTM, with no significant differences between the two arms. However, interns adopting NTM reported that their clinical skills improved more significantly than those of TTM, likely due to the closer patient interactions. Regarding the self-perceived cultivation of clinical thinking, although the NTM group scored higher, the difference did not reach statistical significance. This suggests that subjectively, interns did not feel an overwhelming shift in their own clinical reasoning capabilities during the short 2-week internship. Conversely, objective scores for doctor-patient communication abilities were significantly higher, owing to the interns’ increased proximity to patients. Ultimately, the overall evaluation of NTM by interns was significantly more favorable, indicating a stronger preference for the novel approach. This superior acceptance and favorable perception closely echo recent findings in urological education indicating that trainees preferentially interact with educational resources and digital materials tightly aligned with actual clinical practice and surgical workflows¹⁶. By shifting 3D technology to the bedside to deliver practice-oriented learning experiences, the NTM directly leverages this learner preference, highlighting the substantial educational value of clinically relevant training and demonstrating the pivotal role of innovative tools in driving active learner engagement within urological clerkships.

After completing the questionnaires, a standardized thematic practical examination using standardized wards and standardized patients was conducted. Interns were scored by experienced clinical doctors serving as examiners. TTM and NTM did not exhibit significant differences in professional development with both performing excellent. Interns in the NTM group outperformed their peers in terms of objective clinical standards, skills, and reasoning (*p* < 0.001), which were enhanced by using three-dimensional imaging that overcame the limitations posed by static two-dimensional visualization. Interestingly, in stark contrast to their subjective self-assessment, the objective clinical practice evaluations revealed a different reality. When assessed by experienced examiners utilizing standardized rubrics, NTM interns significantly surpassed TTM interns in actual clinical reasoning and thinking. This discrepancy indicates that while the patient-specific 3D bedside teaching effectively cultivates higher-order clinical decision-making skills, students may not immediately recognize this internal cognitive growth themselves. Results indicated that interns utilizing the novel teaching mode demonstrated markedly better performances than those following the traditional teaching model. These findings imply that the novel teaching model can capably enhance interns’ clinical competencies and professional qualities.

In the final theoretical knowledge test, both TTM and NTM interns accomplished exemplary results. However, NTM interns had a more excellent score compared to their TTM fellows. This indicates that both teaching modes fulfilled interns’ learning needs for clinical theoretical knowledge. The greater acceptance of NTM and its tighter integration with clinical practice likely consolidated their theoretical understanding, thereby achieving remarkable mastery among the NTM interns.

When interpreting the superior performance of the NTM group, several alternative pedagogical explanations and confounding variables must be carefully considered. First, a “novelty effect” (akin to the Hawthorne effect) cannot be entirely ruled out; the introduction of interactive 3D digital tech tablets may have temporarily triggered heightened student curiosity and engagement, artificially inflating short-term satisfaction scores. Second, the potential influence of increased teacher enthusiasm warrants caution. Instructors tasked with deploying an innovative, technology-driven teaching model may naturally exhibit higher pedagogical enthusiasm, structured preparation, and student investment compared to those delivering routine, traditional ward rounds. Lastly, the unique motivational impact of direct patient contact played a critical role. By anchoring the 3D interactive models in the specific, live patients currently under the interns’ clinical care, the NTM fostered a state of “situated learning” and enhanced empathy. This real-world relevance served as a powerful psychological catalyst, boosting learning motivation far more effectively than the abstract, static case descriptions used in the traditional approach.

Our research has some limitations. First, the inclusion of 128 interns represents a convenience sample from a single academic year at a single center, and no a priori sample size calculation was performed before the intervention. However, a post-hoc power analysis based on the primary composite score outcome (Cohen’s d = 1.09, α = 0.05) revealed a statistical power exceeding 99%, indicating that the sample size was highly adequate to detect the observed differences. Furthermore, the short training duration underscores the need for future multi-center cohorts with long-term interventions. Second, it was impossible to blind students to their teaching interventions. NTM students were aware they were receiving a novel approach, which may have introduced the Hawthorne effect, potentially artificially inflating their subjective satisfaction on the confidential questionnaires. Third, while the theoretical exams were blindly graded, complete blinding of the clinical examiners during the practical assessments was challenging. The assessment instructors were frontline clinical doctors who had professional relationships with the interns and might have been aware of their group allocations. Although we rigorously utilized Bloom’s Taxonomy as an objective grading rubric to mitigate subjective inflation, this proximity and lack of blinding may still have influenced the final assessment outcomes. Crucially, complex professional domains involving interpersonal communication and live clinical reasoning remain inherently susceptible to subtle observer influence and expectancy bias, even when structured, objective evaluation rubrics are systematically applied. Additionally, we performed multiple non-parametric comparisons across various sub-domain items without applying explicit multiple testing corrections, such as the Bonferroni adjustment. Consequently, readers are reminded that some secondary outcomes demonstrating borderline statistical significance may not remain significant after formal adjustment procedures; the interpretation of these findings should therefore remain cautious. Specifically, secondary sub-domains hovering close to the alpha threshold—such as clinical standards and skills (*p* = 0.031) and communication competencies (*p* = 0.046)—must be interpreted with appropriate prudence, as strict multi-testing corrective models could attenuate their statistical significance. Nevertheless, our primary endpoints—including the overall composite scores, theoretical examination scores, and critical competencies like clinical thinking—demonstrated highly robust differences that easily withstand conservative adjustments, affirming the overall pedagogical efficacy of the NTM. To address these limitations, we plan to conduct further multi-center studies in future research, expanding the sample size and employing a mixed evaluation method across multiple centers to confirm and extend our findings.

## Conclusion

In conclusion, the integration of patient-specific three-dimensional interactive digital models with real-world case-based bedside teaching represents an effective pedagogical advancement in undergraduate urological training. The empirical evidence demonstrates that the NTM significantly enhances short-term academic performance, objective examination scores, and critical clinical reasoning skills among medical interns compared to the traditional approach. However, our findings do not establish a direct link to long-term knowledge retention, systemic clinical competence, or direct patient outcomes. Future multi-center, longitudinal studies with extended follow-up periods are strictly warranted to fully determine the long-term educational sustainability and patient-level clinical benefits of this novel teaching framework.

## Supplementary Information

Supplementary Material 1.

## Data Availability

The data used in the present study could be available by contacting the corresponding author if necessary.
