# Supplementary material for: Development and implementation of a novel clinical teaching model integrating real-world case with patient-specific three-dimensional imaging in urology
Source: BMC Med Educ. 2026 Jul 1;26:1199. doi: 10.1186/s12909-026-09765-9 (PMC13393478; doi:10.1186/s12909-026-09765-9)
Supplement: Supplementary file 1 — Supplementary Material 1. [file 12909_2026_9765_MOESM1_ESM.docx]

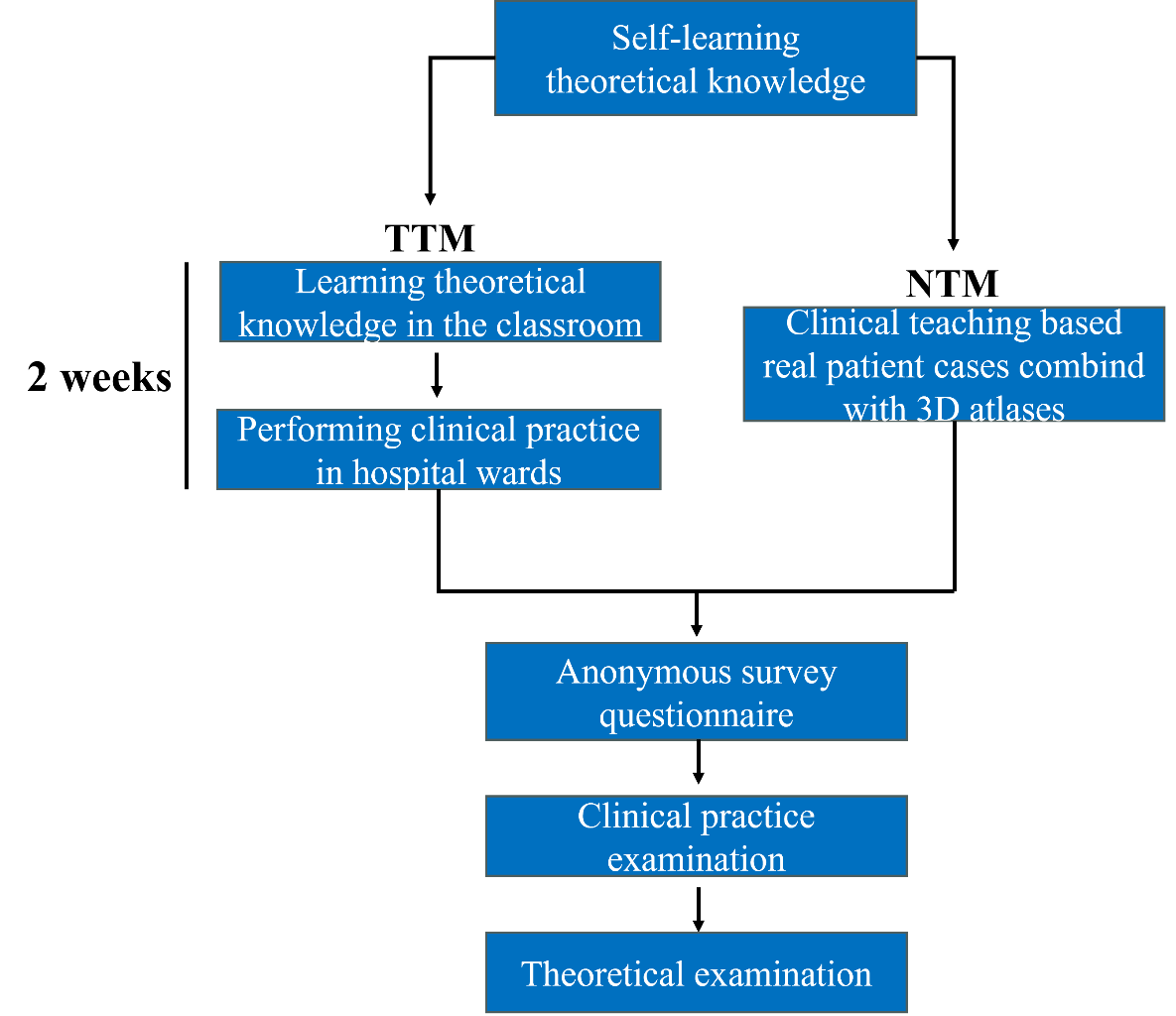


**Figure S1.** Overall flowchart of urology internship.

**Table S1. Anonymous Subjective Questionnaire (5 questions)**

| Question | Options |
| --- | --- |
| 1. Your overall evaluation about these courses: | ☐Dissatisfied （0）☐Moderately dissatisfied （5）  ☐Difficult to judge （10）☐Satisfied （15）☐Very satisfied（20） |
| 1. This teaching mode helped you to understand and master the basic and clinic knowledge: | ☐Small（0） ☐Moderate, indifferent （5）  ☐Difficult to judge （10）☐Large （15）☐Very large（20） |
| 3. In relation to improving your clinical skills in this teaching mode, your learning was: | ☐Small（0） ☐Moderate, indifferent（5）  ☐Difficult to judge（10） ☐Large （15）☐Very large（20） |
| 4. In relation to training your clinical thinking ability in this teaching mode, your learning was: | ☐Small（0） ☐Moderate, indifferent（5）  ☐Difficult to judge（10） ☐Large（15） ☐Very large（20） |
| 5. In relation to cultivating capability of patient-doctor communication in this teaching mode, your learning was: | ☐Small（0） ☐Moderate, indifferent（5）  ☐Difficult to judge （10）☐Large（15） ☐Very large（20） |

**Table S2. Departmental Rotation Examination Score Sheet**

| Items | Detailed Grading Rules | Scores |
| --- | --- | --- |
| Possessing Professional Accomplishment | Intern doctors are compassionate, responsible and altruistic, and fulfill the "patient-centered" medical philosophy. | ☐20 ☐15 ☐10 ☐5 ☐0 |
| Gaining Knowledge and improving Clinical Skills | Intern doctors have evidence-based medicine thinking. according to professional guidelines, following the best evidence, they can combine with clinical experience and patient needs, balance, select and implement rational diagnosis and treatment decisions.  Intern doctors master the clinical skills required by the specialty and have ability to practice medicine independently in this specialty. | ☐20 ☐15 ☐10 ☐5 ☐0 |
| Developing Independent Clinical Thinking | With the guarantee of patients' medical safety as the core, intern doctors can use their professional abilities to develop individualized diagnosis and treatment plans and provide effective and appropriate medical care services. | ☐20 ☐15 ☐10 ☐5 ☐0 |
| Promoting Interpersonal Skills | Intern doctors have humanistic feelings of clinical communication ability, to establish mutual trust and harmonious doctor-patient relationship, to solve practical clinical problems. | ☐20 ☐15 ☐10 ☐5 ☐0 |
| Self-improving Capability | With the concept of independent learning and lifelong learning, intern doctors take the initiative to use various academic resources to continuously track medical progress, update medical knowledge and concepts, and carry out or participate in scientific research based on clinical problems and needs. | ☐20 ☐15 ☐10 ☐5 ☐0 |
|  |  |  |
